# Wireless Power Transfer in Wirelessly Powered Sensor Networks: A Review of Recent Progress

**DOI:** 10.3390/s22082952

**Published:** 2022-04-12

**Authors:** S. M. Asiful Huda, Muhammad Yeasir Arafat, Sangman Moh

**Affiliations:** Department of Computer Engineering, Chosun University, 309 Pilmun-daero, Gwangju 61452, Korea; shamsasif@chosun.kr (S.M.A.H.); myarafat@chosun.ac.kr (M.Y.A.)

**Keywords:** wireless power transfer, SWIPT, energy harvesting, wirelessly powered sensor networks

## Abstract

With the emergence of the Internet of Things (IoT), billions of wireless devices, including sensors and wearable devices, are evolving under the IoT technology. The limited battery life of the sensor nodes remains a crucial implementation challenge to enable such a revolution, primarily because traditional battery replacement requires enormous human effort. Wirelessly powered sensor networks (WPSNs), which would eliminate the need for regular battery replacement and improve the overall lifetime of sensor nodes, are the most promising solution to efficiently address the limited battery life of the sensor nodes. In this study, an in-depth survey is conducted on the wireless power transfer (WPT) techniques through which sensor devices can harvest energy to avoid frequent node failures. Following a general overview of WPSNs, three wireless power transfer models are demonstrated, and their respective enabling techniques are discussed in light of the existing literature. Moreover, the existing WPT techniques are comprehensively reviewed in terms of critical design parameters and performance factors. Subsequently, crucial key performance-enhancing techniques for WPT in WPSNs are discussed. Finally, several challenges and future directions are presented for motivating further research on WPSNs.

## 1. Introduction

In light of the enormous breakthroughs in wireless technology over the last decade, various types of applications based on the Internet of Things (IoT) have dominated our daily lives, such as healthcare, communication, transportation, and intrusion detection [1]. Considering the critical perspective of these applications in terms of security and safety, ensuring connectivity among the nodes is a prerequisite. Accordingly, this can be achieved by ensuring that every node is connected to at least one neighboring hop, or by providing a power supply to the wireless nodes so that these sensors can operate without any interruption [2].

Currently, there are many potential solutions to recharge these sensors. First, the sensors can be connected using wires to a power source. Second, the sensors can be charged with power sources, such as batteries. However, batteries have a limited lifetime, which is one of a primary drawbacks of being connected to sensor networks [3]. To address the issue of uninterrupted power supply, the wireless power transfer (WPT) of charging devices can eradicate the issue of interruption in wireless sensor devices and maintain connectivity to ensure better network lifetime and throughput. Furthermore, with the growing number of wireless devices, the power supply without any interruption becomes increasingly complicated since it requires higher maintenance costs because of the traditional method of charging these devices, either by changing the battery or utilizing wired charging [2]. This becomes even more difficult in remote areas, where natural resources are scarce. In this sense, harvesting energy via wireless devices can address this problem if radiofrequency signals are sufficiently available [4]. Utilizing the characteristics of microwave wireless power transfer, wirelessly powered sensor networks (WPSNs) have several advantages, such as serving a stable power supply and reducing maintenance costs. With the aid of wireless energy harvesting, sensor nodes can have mobility and can be fixed in walls or even in bodies without having their abilities hampered [2].

Despite significant performance improvements, constructing an efficient WPSN remains a challenging task in practice. As charging is performed wirelessly, the sensor nodes may receive lower energy than they should because the wireless devices are located far from the energy transmitters. Moreover, this can affect performance at several locations [5]. Another significant drawback is that it satisfies the requirements of the amount of transferable power and actual power requirement. These issues motivate the consideration of designing the information transfer and the wireless energy transfer jointly. In addition, there are some drawbacks in terms of the communication channels. During energy transfer, there is a possibility that it may share the same spectrum as the communication channel. This requires the construction of data and energy transmission techniques to increase the effectiveness of WPSNs [6].

### 1.1. Related Surveys

In the last few years, numerous surveys have comprehensively summarized existing WPT technologies. Most existing surveys treat the various types of network models separately. Even though they are related to our study, some are out-of-date, and others are not closely related to WPSNs. In this paper, therefore, we focus on the recent progress of the WPT targeted to WPSNs. The comparison of our study and the existing surveys related to our study is summarized in Table 1, in which the distinguishable features of our work are addressed in the last row.

In [7], the authors studied the historical development of wireless power transfer technology and discussed three categories of WPT in terms of architecture, advantages, limitations, and plausible limitations for WSNs. However, in this study, the authors did not discuss the issues that are prevalent in the implementation of these technologies. The authors in [8] provided a brief overview of multiantenna-based wireless information and power transfer (WIPT) and the performance metrics related to the implementation of WIPT. The authors then classified WIPT into wireless-powered communication and simultaneous wireless information and power transfer, which was followed by a brief discussion of these two models and the integration of these two techniques for large-scale multiple-input multiple-output (MIMO) systems. Moreover, in [9], the authors presented a comprehensive survey on state-of-the-art wireless charging technologies, along with the application perspective of wireless communication networks. In [10], the authors studied the historical development of WPT, followed by a fundamental overview and the technical details of its implementation in biomedical devices. Moreover, they also emphasized the simultaneous wireless information and power transfer from the implementation perspective of implantable biomedical devices.

The authors of [11] focused solely on wireless-powered communication networks (WPCNs) by providing background knowledge, enabling technologies, and future research directions in the field. In particular, three major performance-enhancing techniques were focused on in light of the existing literature. In [6], the authors provided a similar review in which they discussed several techniques to improve the performance of WPCNs. However, these two articles focused on communication networks without considering other wireless power transfer techniques. In [12], the authors provided a comprehensive review of simultaneous wireless information and power transfer (SWIPT). The survey covered the foundational aspects of wireless power transfer and radiofrequency energy harvesting, from both the academic and industrial aspects of SWIPT. Moreover, the authors also proposed some insightful research directions. The authors in [13] covered recent advancements in SWIPT, focusing on the aspects of IoT devices from both the radiative and reactive perspectives. In [14], the authors presented a review focusing on the radio regulations of wireless power transfer via microwaves from a historical perspective. Moreover, the authors in [15] provided a historic overview of both WPT and WIPT by discussing state-of-the-art methods and the fundamental building blocks of WPT and WIPT. A review of intelligent reflecting surface (IRS)-aided WPT and SWIPT systems was provided by the authors in [16].

**Table 1:** Comparison of the existing surveys related to our study.

Paper	Year	Target Systems	WPT	SWIPT	WPCN	Key Points
[7]	2013	WSNs	√	🗴	🗴	History of WPT Review of pros and cons on existing WPT technologies Suitability of WPT in WSNs
[8]	2015	Large-scale MIMO and full-duplex systems	√	√	🗴	Main technologies of WIPT Comparison between multi-antenna-based WIPT techniques
[9]	2016	WSNs	√	🗴	√	History of wireless charging research Fundamental wireless charging technologies Wireless charger scheduling strategy Wireless charger dispatch and deployment strategies
[10]	2017	WSNs	√	√	🗴	History of near-field magnetic WPT and communication in free space Review of near-field wireless power transfer and magnetic communication in biomedical systems Near-field magnetic-based SWIPT
[11]	2017	Wireless networks and cellular networks	🗴	🗴	√	Fundamental overview and architecture of WPCNs Approaches and implementation perspectives for WPCNs
[6]	2016	WSNs	🗴	🗴	√	The basic architecture of WPCNs Performance-enhancing techniques in WPCNs
[12]	2018	General wireless communication systems	√	🗴	√	Fundamentals of radiofrequency energy harvesting Fundamentals of WPT and SWIPT techniques SWIPT-enabled communication technologies
[13]	2021	WSNs	🗴	🗴	√	Fundamental overview of SWIPT and WPT Reactive SWIPT for application in the power electronics industry Radiative SWIPT for low-power WSNs in the IoT world
[14]	2020	Not specified	√	🗴	🗴	History, key technologies, innovation, and regulation status of wide- and narrow-beam WPT
[15]	2022	Low power sensor devices	√	√	🗴	History, overview, state-of-the-art technologies, and building blocks of WPT and the wireless transfer of information and power
[16]	2022	IoT applications	🗴	√	√	State-of-the-art techniques on IRS-aided WPT and SWIPT systems
Our work		WSNs and modern wireless communication systems	√	√	√	Application scenario of WPT in sensor networks Review of the fundamental building blocks of WPSNs Classification of WPSN techniques Review of the main techniques for enhancing energy efficiency Enabling analytical frameworks Propose future research directions

As discussed above, existing surveys emphasize wireless power transfer techniques separately from the perspective of WPSNs. In contrast to existing surveys, we discuss all three network models of WPSN: WPT, SWIPT, and WPCN. Furthermore, we provide an overview of the techniques that enable the corresponding architecture. In addition, we compared the techniques in terms of their advantages, limitations, outstanding features, and optimization objectives. Moreover, we provide the key techniques and enabling frameworks that enhance the energy efficiency of WPSNs. A detailed summary of the contributions of this study is provided in the following subsection.

### 1.2. Contribution of This Study

This study aims to investigate the present network models of WPT by reviewing the enabling techniques to build a WPSN. The key contributions of this study are summarized as follows:

- A brief overview of the motivating application scenario of wireless power transfer techniques in WPSNs is provided. The architecture and fundamental building blocks of a WPSN are also reviewed.
- The existing WPT techniques are classified into three network models: WPT, SWIPT, and WPCN. Subsequently, twelve techniques are overviewed and addressed in terms of the basic operational strategy.
- Then, the twelve techniques are comparatively discussed in depth in terms of the main idea, advantages, limitations, performance-centric objective, considered metrics, and outstanding features to provide an idea of selecting the appropriate technique for applications.
- Finally, the crucial techniques for enhancing WPT efficiency and the enabling frameworks for WPT are discussed to enhance the performance of WPT in WPSNs. In addition, some new challenges and future research directions are presented to motivate further research efforts in WPSNs.

### 1.3. Organization of This Paper

**Figure 1:**
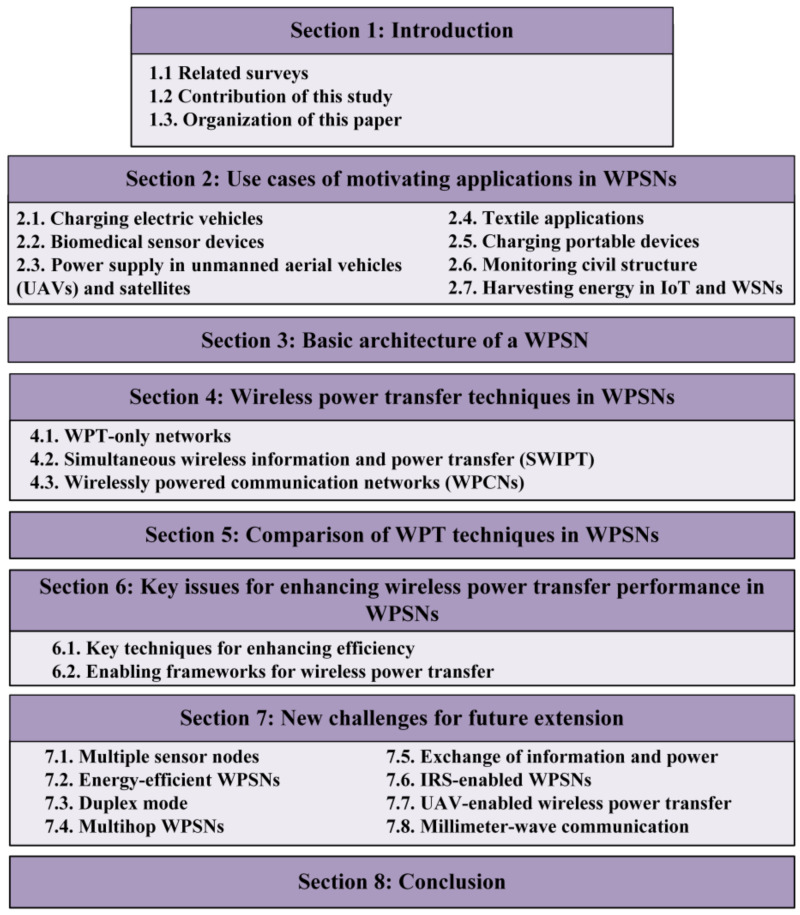
Outline of the survey.

As shown in Figure 1, this survey comprises eight sections. In Section 2, a motivating application scenario is provided. In Section 3, the overview of the basic building blocks of WPSNs is discussed. In Section 4, the existing WPT techniques are categorized and extensively reviewed. In Section 5, the twelve techniques are reviewed in terms of their advantages, limitations, and design goals. In Section 6, the main techniques for enhancing WPT efficiency, as well as the frameworks that enable the study of WPSNs, are discussed. In Section 7, some open challenges for future extensions are provided to motivate further research in this field. Lastly, the paper is concluded in Section 8.

## 2. Use Cases of Motivating Applications in WPSNs

**Figure 2:**
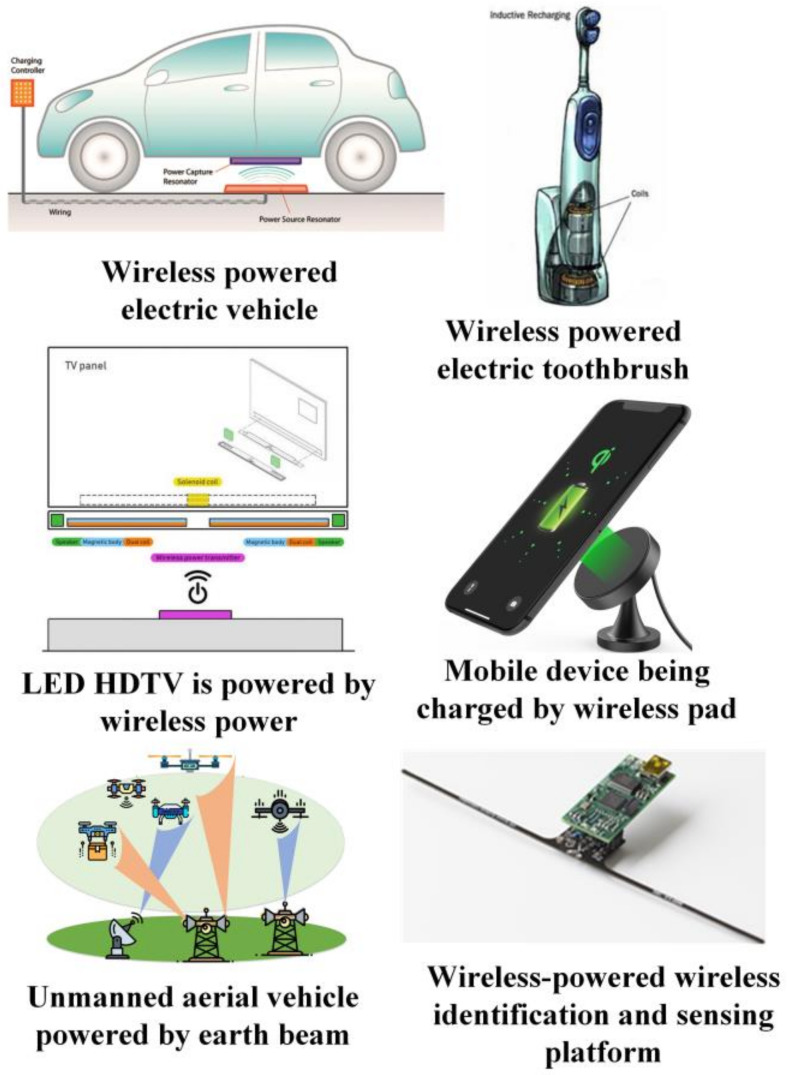
Applications of WPT technologies in WPSNs [7,19].

Wireless sensors are widely used for various purposes, such as monitoring and tracking in environmental and urban areas. With the immense growth of IoT devices in the future, eventually, billions of devices in the form of wireless sensors will be active, which will be the next evolution in WSNs. It will open a new dimension from the perspective of smart cities, homes, and healthcare monitoring systems. Transferring a specific amount of energy from a dedicated energy source to the device widens the potential application scenarios [17]. As shown in Figure 2, we are going to provide a variety of application scenarios in this section to help the readers gain a clear understanding of how wireless power transfer is utilized in several aspects. This covers various types of scenarios from a wide range of perspectives, such as healthcare, surveillance, monitoring, and smart cities. Note that this section restricts its discussion to application scenarios, while other perspectives, such as challenges, will be broadly discussed in the later sections.

### 2.1. Charging Electric Vehicles

In recent years, the technological shift from traditional combustion to electrical engines has brought about a drastic transformation in the car industry. The introduction of WPT has solved the challenges associated with prolonged charging times and deployment costs. Recently, an emphasis has been placed on the enhancement of dynamic charging technologies for allowing power transfer in moving vehicles, as well as a connected part of the vehicle-to-grid concept. Recent studies [18] report that cars and trains can achieve 20 kW and 200 kW, respectively, despite the challenges faced by air gaps with near-perfect accuracy. Roadway-powered electric vehicle systems (RPEVs) refer to wireless power supplied by roadside infrastructure in motion, another area that has seen the light of development. The Korea Advanced Institute of Science and Technology took the first initiative in 2013. A total distance of 2.4-km of roadway includes cables and coils under the surface.

### 2.2. Biomedical Sensor Devices

Generally, in the case of implant devices, energy harvesting is critical because of the risk of complications, imbalanced voltage supply, and limited lifetime. However, comparative studies between inductive power transfer and radiative power transfer [20] reveal that radiofrequency-enabled systems can enhance the overall performance in such a scenario. Even if the present studies prove the successful implementation of cardiac and retinal neuromedical sensors, another study concluded that the efficiency falls short in terms of safety and poor effectiveness, which are expected to diminish with advances in transmission optimization and miniaturization [21].

### 2.3. Power Supply in Unmanned Aerial Vehicles (UAVs) and Satellites

The power supply is of crucial importance in UAVs and satellites to ensure continued reliable performance. Therefore, the weight is maintained as light as possible. The fundamental idea of transmitting power in such a scenario is that the receiver is tracked to ensure a continuous supply, whereas the transmitter often remains stable. Moreover, transferring power in the opposite direction and bidirectional communication are possible. As presented in [5], the inclusion of WPT enables UAVs to utilize downlink communication as the distribution of optimized energy. In addition, a WPCN can be formed by enabling the UAV to act as a mobile base station [6,12].

### 2.4. Textile Applications

Charging devices wirelessly is a crucial challenge to ensure the continuous monitoring of on-body monitoring devices. Therefore, it is mandatory to integrate the coils into textiles to enable such a phenomenon. Manual replacement is required while washing such textiles, which is inconvenient. Recent studies have shown that textiles can be integrated with energy storage devices, such as textile supercapacitors [22] and secondary batteries [23], which require charging. The integration of such devices must be performed cautiously such that the characteristics remain intact and unaffected. In [24], the authors discussed several fabrication techniques for integrating coils into flexible textiles.

### 2.5. Charging Portable Devices

Nowadays, the use of portable devices, such as smartphones, tablets, and smartwatches, has increased unprecedentedly. With the increase in various functionalities in such small devices, power consumption and charging for the least possible time have recently become very demanding.

With the introduction of WPT, the traditional charging system can be replaced, which enables the continuous charging of a laptop or smartphone without the need to plug in as in wired charging. In [25], the authors provided a comprehensive study on the fundamental topics and underlying physics of wireless charging techniques to facilitate a better understanding of the application of wireless charging for any device. The authors also discussed distributed wireless charging for mobile devices and proposed two wireless charging architectures.

### 2.6. Monitoring Civil Structure

During construction, a major duty involves monitoring a large structure to prevent unexpected events. Moreover, monitoring may also be required throughout the lifetime to ensure the prediction of structural weakness. In such scenarios, wired sensors are widely used despite their complicated implementation. Moreover, battery replacement is another critical issue for long-term deployment. To solve this issue, the work in [26] demonstrated a WPT method that is integrated deep into concrete. In particular, they designed a system in which all nearby structures are made of steel bars, known as rebars, which can act as a magneto-inductive field. The research outcome shows that the sensors can be powered at distances of approximately 700 mm.

### 2.7. Harvesting Energy in IoT and WSNs

With an increase in the number of IoT devices in the near future, there will be a drastic change in the IoT industry. More specifically, billions of devices will be connected to perform computationally intensive tasks (e.g., surveillance, road, environment safety, augmented reality, and healthcare). However, a continuous supply of energy is crucial to achieve future goals, primarily because most IoT devices have a limited battery lifetime. In such cases, WPT is a promising solution because it eliminates the need to replace the conventional battery and reduces the cost by a significant margin. In wireless sensors, there are three principal ways in which energy is consumed, which are presented in [27] as:

1. Energy involved in transmitting and receiving radiofrequency. Because both the energy transmitter and receiver consist of RF components (e.g., analog-to-digital converter (ADC) and mixer), these components require energy during transmission and receiving the energy signal at/from a certain distance.

2. Energy involved in information processing. The sensing module senses the information, which consists of a sensing chip along with an ADC.

3. Energy involved in the active mode refers to the energy consumed by the internal processing unit of the sensor node. Generally, the processing unit consists of a very small storage unit that executes the task, completes data processing, and maintains the functionalities of the different components present in the sensor node.

A wide range of approaches have been proposed in the literature to reduce the energy consumption of IoT devices during the three active modes. These include scheduling tasks [28], dynamically managing power [29], and optimizing control of the computing unit CPU cycle [30,31]. It was observed that the energy consumption involved in transmission was much larger than the energy consumption involved in sensing and processing. Needless to say, most of the current literature focuses only on the energy cost of transmission, excluding the energy incurred in sensing and processing [32,33]. However, based on the application, IoT may require more complex sensing functions, such as complementary metal–oxide–semiconductor (CMOS) image sensors and seismic sensors [34]. In such cases, the scenario may change, and the energy cost involved in processing could be much higher than that of the transmission task. In [35], the authors considered the energy consumption related to sensing and transmission to solve the energy allocation problem.

## 3. Basic Architecture of a WPSN

Wireless sensor networks (WSNs) supply various benefits, such as remote sensing, data collection, surveillance, and fire/flood emergencies, without deploying any wires, which also saves human labor and increases productivity. The source of power is mainly a disposable battery for wireless sensors. Due to the limited battery lifetime of the wireless sensors, the maintenance of large-scale WSNs becomes a huge burden. Despite the possibility of changing the battery periodically, this becomes impossible in complex environments that are either dangerous or complicated [36]. Although the existing studies emphasize power management techniques, the lifetime of sensor nodes remains a drawback that makes the deployment of large-scale WSNs even more challenging. Thanks to WPT, utilizing the ability to power sensor devices, it is now possible to build a batteryless wireless sensor network that is powered wirelessly from the energy source. To deal with the evolving development of sensor networks, TDK developed WPT technology by utilizing magnetic resonance technologies for various industrial applications [37]. Some of the notable WPT technologies developed by TDK are resonated capacitors for matched resonant tuning (achieving high efficiency by using miniaturized magnetic dielectric materials) and optimized power system solutions at different power levels. These technologies provide various facilities, such as power supply, not only with rotating robot arms and surveillance cameras but also with enhanced reliability and safety. In the existing literature, wireless-powered WSNs are considered to be solving the energy-constraint issue in WSNs [38,39]. In [38], the authors proposed a multi-antenna-based wirelessly powered sensor network, where electric energy is transferred from a power station to a sensor node. The authors in [39] demonstrated the power allocation technique using multiple antennas and a random source for distributed estimations.

**Figure 3:**
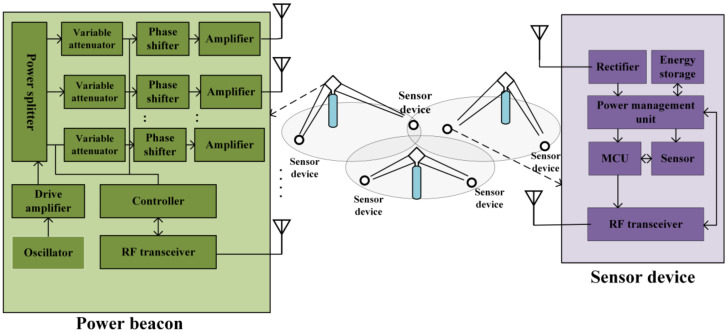
Basic architecture of a wireless-powered sensor network.

The general concept of the WPSN is presented in Figure 3 [40]. Typically, a WPSN model comprises a power beacon broadcasting power to the sensor nodes located in the beacon’s coverage. An antenna array is integrated with the power beacons to enable the power beacon to transmit power through the microwave beam. Beamforming circuits must maintain the phase and magnitude of the signal at an optimal rate to ensure that the microwave beam is adaptively steered. In general, there are two types of antenna array: analog and digital. In an analog array, an oscillator generates a radiofrequency signal. A power divider splits the signal into individual radiofrequency paths. Before the antenna transmits the signal, the signal is passed through a variable attenuator, phase shifter, and an amplifier. Collectively, the variable attenuator and phase shifter cause changes in the magnitude and phase. The digital antenna array consists of a digitally modulated signal generator. The digital modulator includes a digital-to-analog converter (DAC), a filter, an amplifier, and a mixer. The baseband signal emitted from the DAC is transformed into a radiofrequency (RF) signal using a mixer.

When selecting the beamforming circuit, the requirements of radiofrequency wireless power transfer must be considered. Antenna arrays with a large number of antenna elements must be within a reasonable cost. Because the digital array requires a DAC converter, it is very costly to implement, although it provides more versatility than the analog array.

Now, the basic building blocks of the sensor nodes are introduced. The sensor nodes in the WPSN can be formed by integrating a traditional sensor device with a radiofrequency energy-harvesting circuit. The basic building blocks of energy-harvesting circuits include the following: a receiving antenna, a unit for managing power, and a rectifier. The radiofrequency generated from the antenna is converted to DC power using a rectifier. Generally, there are several components in the power management unit, which are a DC–DC converter, a battery for energy storage, and a portion of power consumption. Typically, batteries can store energy. The power consumption portion consists of a microcontroller unit (MCU), sensors, and a radiofrequency transceiver. The transceiver is simply a chip that implements lower-power communication standards and low-energy Bluetooth. The MCU is a combination of a central processing unit, memory, and peripherals. The MCU collects data from the sensors and sends it to the power beacon with the help of radiofrequency.

The sensor nodes have a limited power source; therefore, the energy consumption model must be optimized to enhance network performance. In addition, sensor nodes mostly perform both information transmission and energy consumption, so it is necessary to jointly optimize both. In a practical scenario, the sensor node’s energy consists of the power consumed by the circuit and the sensing power.

Sensor nodes perform the task of transmitting information to an access point through several techniques, such as energy beamforming [41,42] and backscatter communication [43,44]. Sensor nodes can be equipped with an antenna to perform a range of tasks, such as energy transmission and information transmission [3]. It is crucial to consider the type of channel model that transfers both energy and information; otherwise, the channel interference can affect the overall performance of the network.

Regarding the information transmission from the sensor nodes to the information access point, sensor nodes contain some initial information that can be obtained either by sharing information between the sensor nodes or by measuring the temperature information. It is also necessary to maintain synchronization between the sensor nodes in terms of frequency and time. In a real-time scenario, when the sensor nodes are activated during information transmission, energy is consumed for sensing and also from the circuit.

## 4. Wireless Power Transfer Techniques in WPSNs

**Figure 4:**
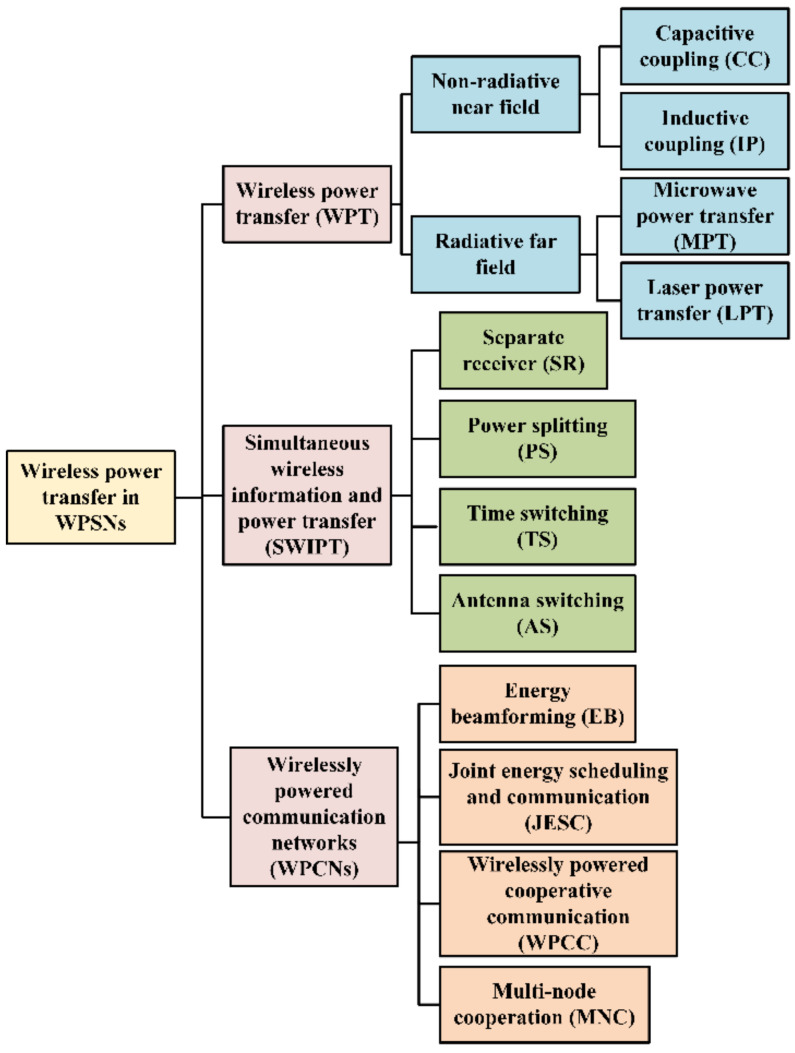
Classification of wireless power transfer (WPT) techniques: CC [45], IP [46], MPT [47], LPT [48], SR [36], PS [49], TS [50], AS [51], EB [52], JESC [53], WPCC [54], and MNC [55].

In this section, we classify existing WPT technologies in sensor networks into three categories based on the basic network architecture, as shown in Figure 4. First, we consider the WPT-only network, where only power transfer is involved in the sensor network. Second, the demand for simultaneously sending information along with energy is indispensable in any network facilitated by SWIPT techniques. Finally, we shed light on WPCN techniques, where the flow of information and energy transmission are managed separately via an uplink and downlink.

### 4.1. WPT-Only Networks

WPT has been dominant in a wide range of applications, such as satellite communication and the identification of radiofrequency tags. In 1890, Nikola Tesla first introduced the concept of WPT. The general concept behind a WPT system is to transmit electric energy from a power source utilizing an electromagnetic field to an electromagnetic circuit, where power is transmitted and received without the need of a wire. The system consists of a single or multiple power beacons and energy receivers. Generally, the sensor devices are powered by randomly distributed power beacons that are received by energy receivers. It allows the sensor device to overcome the battery bottleneck with an extended battery life and to overcome the battery replacement of the sensor nodes. The fundamental concept of wireless power transfer is to transform energy into an electromagnetic field, which is then transmitted to the energy receiver. The transmission of power over long distances and high-power applications are two factors that play a critical role in the performance of WPT [12]. The existing literature has vastly focused on WPT techniques in sensor networks. The authors in [56] emphasized the age of information as a metric for a sensor network with WPT capability. The authors considered the importance of freshness of the received information by formulating a simple optimization problem. In [41], the authors demonstrated a WPSN where sensor nodes transmit information using wireless energy transferred from a group of energy transmitters to an information access point situated far away. Unlike these, the authors in [57] proposed a scheduling strategy to maximize the data packet loss in a sensor network with respect to the nodes’ energy consumption and data queue information. Considering the distance between the energy transmitter and the receiver, existing WPT techniques can be classified into two categories: non-radiative near-field and radiative far-field. Next, we provide a brief overview of these concepts. In Table 2 and Table 3, we summarize different WPT techniques in terms of their advantages, limitations, and operational characteristics.

#### 4.1.1. Non-Radiative Near-Field

Near-field refers to the electromagnetic field regions that are close to the object. The near-field technique is mostly utilized to harvest energy at a shorter distance of a few centimeters between the energy transmitter and receiver. The fields in this zone are very strong, are very close to the antenna, and are suitable for applications such as radiofrequency tag technology and wireless charging. With an increase in distance, the field strength sharply decreases, which affects the overall energy transmission. Since the WSN lifetime is constrained by the limited battery lifetime, WPT techniques including near-field techniques can be beneficial for WSNs in several ways. For example, in planar applications (e.g., mobile phone pads), this technique can ensure efficiency and short air gaps. Inductive coupling is also suitable for mid-range applications. Recharging the sensor nodes remains a burden despite the recent developments in battery technology. To charge the batteries of wireless sensors deployed in a large area, drones are being used, which have a limited battery as well. In such a scenario, inductive coupling techniques can be used for wirelessly charging the UAV battery, which charges the sensor nodes using the same method. Thus, the communication range can be expanded over a large area [60]. In [7], the authors demonstrated the three categories of WPT, where they analyzed and discussed the scope of WPT technology in sensor networks. To be specific, the authors concluded that magnetic resonant coupling is more suitable for WSNs compared to other techniques. Typically, non-radiative near-field techniques can be classified into two types, which are capacitive coupling and inductive coupling. To get an idea of the near-field techniques, a brief overview of these two techniques is provided in the next subsection.

##### Capacitive Coupling (CC)

In capacitive coupling WPT, energy is transferred between the two blocks of the circuit via a capacitor. A coupling capacitor is placed between two blocks that transmit the AC signal and block DC energy. The coupling functions as a medium to omit the DC signal. The coupled capacitor provides sufficient resistance to the DC voltage and, simultaneously, a comparatively lower resistance to the AC signal. The unavailability of any receiving device within the couple range results in no power leaving the transmitter. The performance can be improved by either scaling up the operating frequency, such that the impedance of the circuit is reduced, or a compensation inductor can be utilized to neutralize the capacitive impedance. The primary advantages of this technique include its cost-effectiveness, light weight, and the capacity to be utilized at a short distance [61]. In [45], the authors presented an approach for transferring power using a metal barrier. The authors divided the system into two parts: the transmitter and receiver sides, placing a metal barrier in the middle. On the transmitter side, the DC voltage was transformed to high-frequency AC voltage. This resulted in alternative voltage stimulated between two transmitting plates. Afterward, across the metal barrier, an AC current flowed between both coupling areas, since the transmitting plates were coupled with the metal barrier in the middle. Combining the metal barrier with the transmitting circuit enabled the generation of a magnetic field generator. Its functionality worked in a similar fashion to a traditional inductive power transfer transmitting circuit. The multiple coils in the traditional inductive power transfer system were replaced with the metal barrier, which acted as a primary single-turn coil. The authors demonstrated that, despite the sensitivity around the edge, the output power was higher around the edge of the metal barrier.

##### Inductive Coupling

Inductive coupling is one of the most widely used techniques in the field of WPT and is utilized in almost all commercial products. This technique is based on Ampere’s circuital law and Faraday’s law of induction. Ampere’s circuital law refers to the link between an integrated magnetic field and the electricity passing across the loop. Faraday’s law refers to relation between an induced magnetic field and a time-varying magnetic field. The efficiency of the WPT largely relies on the coupling coefficient, which depends on the two-coil distance as well [62]. In inductive coupling, a transformer-like model is designed and implemented by combining transmitter and receiver coils [7].

The fundamental operating principle of inductive coupling is like that of a basic transformer. A magnetic field is generated by applying an inductive power transmitter to the wired coil. Simultaneously, the AC voltage is formulated at the receiver end based on the magnetic induction principle. However, due to the air gap characteristics of the transformer, it sometimes results in a very poor magnetic conductive environment. Furthermore, the distance between the coupling devices is also a crucial metric. In the case of a longer distance, this inductive coupling technique is very inefficient, and it causes higher energy consumption in the primary coil resistors [19]. Inductive coupling is one of the oldest types of WPT that is used in real applications. In near-field WPT systems, resonators play a critical role. Recently, a great number of WPT systems have been investigated to enhance the WPT performance. Some of the most well-known resonators are metallic-coil, shielded-loop resonators and printed-coil resonators [63]. In [63], the authors explained the four-coil wire resonator, which is mostly used in the mid-range WPT systems. In such a four-coil system, the source with a driving coil is utilized to excite the transmitting coil. Similarly, a load coil is loaded in the receiving coil. The purpose of these coils is to enhance the power transfer efficiency of WPT systems.

Owing to the advantages of wireless architecture, this technique is simple, convenient, and safer than other techniques. Commercial products utilizing inductive coupling technology have also seen significant developments, such as mobile device charging pads, electric toothbrushes, and medical devices that replace any biological structure. However, this technique fails to achieve a reasonable performance because the transfer rate falls sharply at even a short distance. The study in [46] demonstrated the design of an inductive power transfer system for powering a drone that runs out of battery. The authors proposed a novel design strategy that can achieve sufficient coupling without affecting the structure of the drone. They designed a lightweight air-core coil by incorporating a load-independent inverter at the end of the transmitter to provide a constant-current amplitude. On the receiver side, the authors considered including a hybrid rectifier, which enables tuning for large changes in power demand. The authors proved the accuracy of their study via computer simulation, as they claimed that an average efficiency of 60% could be achieved. Though efficiency over a long distance is a drawback of inductive coupling for WSNs, performance can be enhanced at further distances by utilizing coupled magnetic resonances [64].

#### 4.1.2. Radiative Far-Field

Far-field is the region where the strength of the electromagnetic wave does not weaken with increasing distance. This region acts as a determining indicator for the performance of this technique. Since the receiver is away from the energy transmitter, attenuation still dominates the overall energy efficiency. The operating process of this technique starts by transmitting power through a radiofrequency source using an antenna. The radiated power is propagated through the air until the matching circuit of the receiver captures it and converts it into electricity by rectifying. Accordingly, the DC output supplies energy to the device storage. It is assumed that a great number of wireless sensors are going to evolve to perform data-gathering tasks from their surroundings in large areas in the near future. Depending on the information collected from the sensors, taking real-time decisions and actions will be easier. However, the limited battery lifetime in the large-scale sensor networks is still a challenging issue due to the processing and link overhead [65]. In the existing literature, far-field WPT systems have been extensively discussed for a wireless sensor network. In [66], the authors presented far-field wireless charging techniques for wireless sensors. The authors in [67] demonstrated an adaptive WPT system based on a far-field technique that can be incorporated into passive WSNs. The authors concluded that simple yet efficient far-field WPT systems can be designed to solve the energy constraint problem in WSNs. Depending on the operational frequency, radiative far-field techniques can be classified into two modes, which are described next.

##### Microwave Power Transfer (MPT)

MPT refers to the idea of transmitting power from the source to the destination in the form of microwave frequency [68]. First, in an MPT system, direct current (DC) is converted into a microwave signal, which is then transmitted to the target destination. Before transmitting the microwave signal, it is passed through a tuner and a directional coupler so that the generated signal is separated according to the direction of the signal propagation. At the receiver end, the antenna receives the microwave signal, which is then converted back into DC power. In [47], the authors investigated wirelessly transferring power via microwave radiation. In particular, they proposed the incorporation of an electromagnetic rectifying surface to rectify microwaves without reflection. It enabled the rectifying surface to receive power without being affected by reflection. The authors concluded that the proposed solution could reach an efficiency of up to 42% over a distance of 20 cm.

##### Laser Power Transfer (LPT)

Laser technology has been gaining popularity in military applications. The concept of LPT is to transform electricity in the form of a laser beam. Several optics shape the laser beam before redirecting the laser wave through a beam director to the receiver. After reaching the receiver, some photovoltaic (PV) cells match the received laser in terms of intensity, and the wavelength transforms the laser back into electricity in a usable form [58]. In [48], the authors introduced a distributed laser-charging-based WPT approach that has the potential to provide an identical experience to Wi-Fi communication. Accordingly, in the proposed model, a laser is generated by the electrical power transmitted from the DLC transmitter. The laser then passes through the air until it arrives at the end of the receiver. At the receiver’s end, an external cavity laser is converted into electricity using a photovoltaic (PV) panel. The authors demonstrated the accuracy of the analytical model by extensive simulation to prove maximum power transmission efficiency.

### 4.2. Simultaneous Wireless Information and Power Transfer (SWIPT)

**Figure 5:**
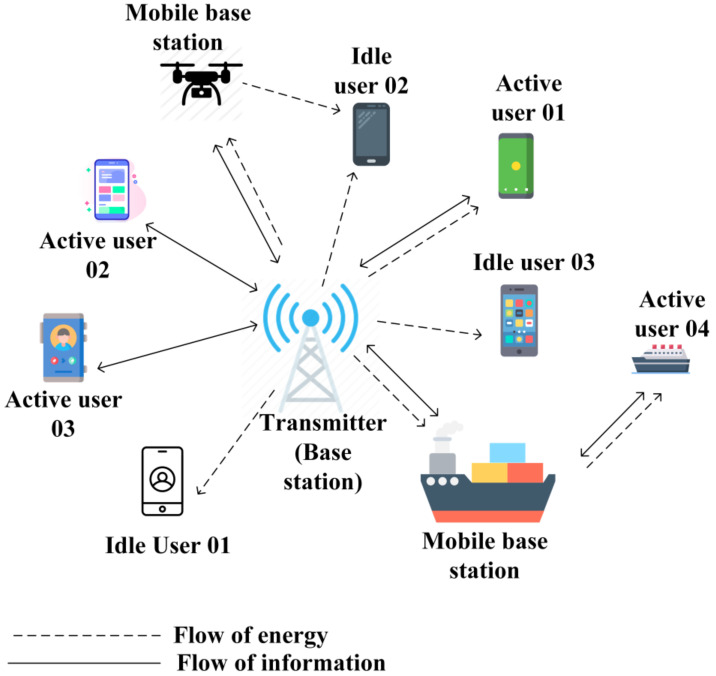
Flow of information and energy in SWIPT using static and mobile base stations.

Unlike WPT-only networks, where only the energy transferred is involved in the entire network, SWIPT facilitates the simultaneous transfer of information and energy using downlink communication, as shown in Figure 5. In Figure 5, the arrowheads correspond to the direction in which information and power flow. Accordingly, idle users can harvest energy from the radiofrequency signals from static and mobile base stations. Meanwhile, the active users transmit and receive information from static and mobile base stations.

**Figure 6:**
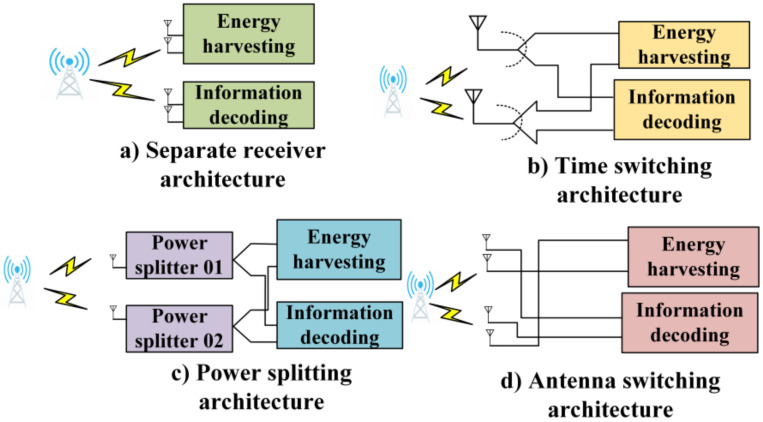
Receiver architecture designs for SWIPT: (**a**) separate receiver architecture with separate receiver for energy and information decoding, (**b**) time switching architecture where time is allocated to the particular antenna for energy harvesting and information decoding, (**c**) power splitting architecture where the power splitting receiver divides the signal into two streams of power depending on power splitting ratio, and (**d**) antenna switching receiver that switches between antennas for energy harvesting and information decoding based on the optimization algorithm.

In SWIPT, a radiofrequency signal that can carry both energy and information is utilized to enable such a phenomenon. The authors of [36] first introduced this technique, where information and energy were simultaneously received from the received signal. In the architecture, the authors utilized two different circuit receivers to perform both information decoding and energy harvesting, because it is impossible to accommodate energy harvesting and information decoding on the same received signal, owing to the possibility that the information content of the signal is affected by the energy-harvesting operation. Therefore, it is necessary to use the received signals separately or to use different antennas with a certain task. Next, SWIPT-enabled receiver architecture will be briefly described, as illustrated in Figure 6.

Figure 6a–d depict a separate receiver architecture, where the energy and information-decoding receivers are separate; time-switching receivers, where a portion of time is allocated to harvest both information and energy; a power-splitting (PS) architecture, where the signal is divided into two streams based on a ratio; and an antenna-switching receiver, where the system switches between decoding information and energy harvesting.

#### 4.2.1. Separate Receiver

In this architecture, as the name describes, two separate receivers have specific antennas [36]. A transmitter equipped with several antennas serves these two receivers to decode information and harvest energy. Moreover, various channels can be observed using these two antennas. Two separate receivers enable this architecture to perform both actions independently. The feedback from the receiver, along with the channel state information, can be utilized to obtain the expected tradeoff between information quality and energy harvesting to further improve performance.

#### 4.2.2. Time-Switching Receiver

Unlike separate receivers, time-switching receivers only have a single antenna that performs both energy harvesting and information decoding. The receiver comprises a radiofrequency energy harvester, an information decoder, and a switch to adjust between different modes. In [36], the authors adopted the time-switching method as one of the two methods in which the receiver can acquire energy and information from the received signal. Depending on the quality of the service and the type of application, the time-switching sequence and transmit signal can be collectively optimized for different criteria. The SWIPT scheme was utilized in [49] to enhance the energy efficiency of the WPSN, where the authors formulated a resource allocation problem. Accordingly, they considered a WSN comprising multiple sensor nodes and one sink. The authors adopted a time-switching receiver design for a node to enable the destination node to serve both as an information receiver and as an energy-harvesting node. The sink was responsible for receiving data and broadcasting energy to all the sensor nodes. The entire sensor node maintained a tree topology during the monitoring task. The authors proposed a schedule initialization algorithm to determine the transmission schedule, along with the allocation of transmit power, data size, and broadcast time, that establishes a conflict-free schedule to achieve maximum energy efficiency.

#### 4.2.3. Power-Splitting Receiver

Power splitting is another technique that simultaneously combines wireless power transfer and information decoding. In this architecture, the received signal is divided into two streams with different power levels. After this stage, the power levels are transferred to the energy harvester and the information decoder. Thus, only the receiver circuit is required to be newly designed in the entire architecture, as the other parts can be used as in the traditional communication system [50].

#### 4.2.4. Antenna-Switching Receiver

Switching between energy harvesting and information decoding is a crucial goal on which system efficiency depends. While some antennas perform a particular type of task, other antennas can perform different tasks, unlike the previous ones. Based on this idea, the antenna-switching technique operates in such a way that, even if some antennas perform information decoding, other antennas can keep performing energy harvesting [51]. This technique is the simplest and the least complex to implement. Subject to a proper antenna-switching approach, this architecture can also be extended to incorporate a large number of antennas. However, in the case of a hardware failure, performance can be affected. Nevertheless, a separate receiver technique can be optimized by utilizing the antenna-switching receiver technique. In Table 4, the existing SWIPT technologies are summarized in terms of the design approach and main idea.

### 4.3. Wirelessly Powered Communication Networks (WPCNs)

**Figure 7:**
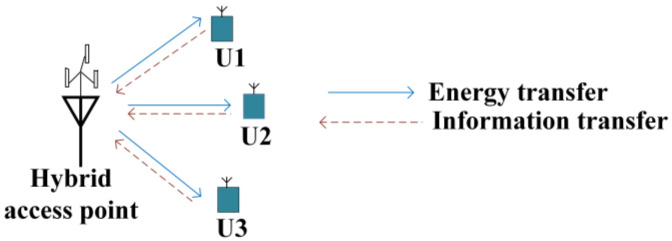
Wirelessly powered communication network with energy transfer and wireless information transmission.

In contrast to SWIPT networks, in WPCNs, energy and information are handled separately by transferring in the downlink and uplink, respectively, as illustrated in Figure 7. In Figure 7, a hybrid access point (H-AP) with a sufficient power supply coordinates the energy and information to/from a few users with limited energy sources [5].

The WPCN eliminates the need for frequent battery replacement by the inclusion of microwave wireless power transfer technology, resulting in improved performance with an extended network lifetime and a lower cost in the communication network, where previous energy was provided by traditional batteries. However, there are still several challenges, such as energy transfer and communication that may occur in the same spectrum, which could result in co-channel interference. In practical applications, the performance of WPCNs is affected by below-average efficiency and the limited range of wireless power transfer, as well as the scarce resources of information and energy transmission. Next, we briefly discuss potential techniques for improving the performance efficiency of WPCNs.

#### 4.3.1. Energy Beamforming

To obtain effective and efficient energy transfer, wireless power transfer requires antennas through which energy can be focused in the direction of energy receivers. The availability of line-of-sight (LOS) links allows for the use of traditional antennas. However, in dynamic environments, such as mobile applications, antenna arrays require handling movements that are achieved through a steerable antenna array, also known as energy beamforming. This technique is comparatively flexible and can more effectively direct energy to a receiver. Precise channel state information between the transmitter and receiver antennas is expected to achieve the maximum power for the receiver. However, when formulating the training procedure, the receiver must consider several parameters, such as duration, transmission power, and frequency bands. The hardware capacity is another challenge that affects the total amount of energy at the receiver. In practice, wireless sensors have a very low computational capacity, which in turn fails to determine the channel state information. A signal that indicates whether to maximize or minimize the power level in every training slot can be adopted to avoid such complexities [52].

#### 4.3.2. Joint Energy Scheduling and Communication

In most cases, the energy scheduling and communication are interconnected. The energy demand of wireless devices is the determining factor based on which the energy transfer protocol should be designed. In contrast, the information transmission capacity cannot be achieved at the maximum level because, at each wireless device, a certain amount of energy is spent while harvesting energy. Such a scenario enforces the joint consideration of energy and communication scheduling to avoid channel interference. However, both communication and energy scheduling can happen simultaneously by equipping multiple antennas in the access point and the energy nodes. The energy nodes can incorporate energy beamforming to steer energy beams directed towards certain users. Access points can adopt space-division multiple access to enable multiple users to transmit on the same frequency resource block [53].

#### 4.3.3. Wirelessly Powered Cooperative Communication

In addition to the previously discussed technique, collaboration between multiple nodes provides another potential solution for enhancing the performance of WPCNs. Accordingly, users can share energy and time resources and collaboratively communicate with access points. For example, consider a scenario in which a high-altitude platform (HAP) serves three users with different energy levels. In this case, the user located at a shorter distance with sufficient energy can assist in relaying the information to the user located farther away. Users located far away may also use the users as a relay to send information back to the HAP. At first glance, it may appear that the near user is consuming more energy, as well as a lower data transmission rate, because it helps the far user. However, this, in turn, favors this user as the collaboration between the users enables the HAP to divide a larger portion of the time in data transmission instead of wireless power transfer [54].

#### 4.3.4. Multi-Node Cooperation

In addition to cooperation between devices, energy nodes and information access points can also cooperate to effectively transmit energy and information. The interconnectivity between the energy nodes and the information access points is ensured by a wired/wireless backhaul link, which enables them to perform such cooperation. More specifically, a multiple-input multiple-output (MIMO) system is formed by the energy nodes in the downlink communication, which enables them to perform distributed energy beamforming to maximize the amount of energy at the target wireless device. Meanwhile, in the case of the uplink transmission of energy, a coordinated multipoint system is built that allows the collaborative decoding of messages received from users. These two events can occur simultaneously, without requiring an individual frequency band. The ability to omit the interference during energy transfer results from utilizing the preset energy signals received from the energy nodes. However, determining the ideal number of energy nodes, the location of the energy nodes, and the access point in a multi-node WPCN are still critical problems that may affect performance in the long run [55].

## 5. Comparison of WPT Techniques in WPSNs

In this section, different WPT techniques are compared with respect to their outstanding features, performance metrics, aims, competitive advantages, and limitations.

In Table 5, existing WPT technologies are compared in terms of their main objectives, advantages, and limitations. Table 6 summarizes the proposed WPT techniques for the evaluated performance metrics, goals, outstanding features, and tools used for simulation. These techniques primarily focus on maximizing wireless power transfer efficiency. Moreover, the tabular study indicates that LPT techniques perform relatively better in terms of the amount of transmitted power in the far-field. This is because of the increased power transfer without any deterioration. In the case of the near-field technique, inductive coupling offers an extended power transfer range compared with capacitive coupling.

Table 7 summarizes the main idea, performance-centric advantages, and limitations of the existing SWIPT techniques, which will enable readers to gain a better understanding of each technique. Table 8 presents a more technical view by comparing the emphasized performance metrics, goal of the study, outstanding features, and tools used in the studies. From the tabular study, it is evident that most studies focus primarily on one performance metric that maximizes the power transmission efficiency considering practical constraints. However, in large-scale sensor networks, maximizing the amount of received power remains an open issue. Moreover, based on the scenario, determining the optimal tradeoff between the information rate and energy transfer is another critical issue that the existing studies are striving to solve because both metrics are equally important. Various constraints have been considered to obtain realistic performance improvements, such as co-channel interference, channel estimation error, and conflicting transmission schedules.

Table 9 presents an overview of the main idea, competitive advantages, and limitations of the existing WPCN techniques considered for this survey. Table 10 summarizes the WPCN techniques from a technical perspective. From the tabular comparison, it can be observed that most studies are targeted at effectively utilizing energy and information transfer by optimizing the total throughput, transmit power, distance ratio, energy-harvesting rate, and deployment cost. Most techniques have been dedicated to improving the total throughput or total sum of energy. It should be noted that, although most studies present extensive simulations to prove the correctness of the model, more realistic constraints should be considered for real-life implementation, such as different tradeoffs between energy and information, co-channel interference, and a large number of users. The design of an effective WPCN technique strongly relies on these factors. Hence, the prevalent issues in WPCNs need to be emphasized to obtain a scalable and highly reliable performance.

## 6. Key Issues for Enhancing WPT Performance in WPSNs

Various aspects must be considered to enhance the performance of WPT in WPSNs. In this section, both the critical techniques for enhancing the WPT efficiency and the enabling frameworks are discussed for WPT in WPSNs.

### 6.1. Key Techniques for Enhancing Transfer Efficiency

Most problems in existing energy-harvesting methods in WSN design are treated as either energy or power scheduling problems. In a single time slot, if the amount of allocated power is too high, it may result in energy disturbance, which would cause the energy-harvesting rate to be very low. Similarly, if the allocated power is too low in a single time slot, energy harvesting can be very high, and consequently, a battery may not be able to store all the energy, resulting in energy wastage. In this subsection, based on the present literature, the techniques to enhance the performance of WPSNs are discussed.

#### 6.1.1. Energy Beamforming

The performance of the WPSN significantly depends on how the energy consumption model appears. Most existing studies focus only on the transmission energy as their total energy consumption. However, in practice, some non-negligible power consumption is related to sensing and circuit power. To solve this problem, [41] considered a network model in which common information is sent to an access point situated far away through several sensor nodes using harvested energy. Using this model, the authors maximized the received signal-to-noise (SNR) ratio at the information access point. Ultimately, they concluded that their system performance significantly improved compared with that of the traditional design. In [38], the authors investigated a WPSN with multiple antennas, where energy was transferred wirelessly to a sensor using an electromagnetic wave.

A smart reflecting surface integrated with a WPSN was introduced in [42] to maximize the performance of energy transfer, as well as information transfer. To achieve this, the authors adjusted the phase shift of the reflecting element in such a way so that the transmission time allocation and phase shift of the WET and WIT were jointly maximized. They started by formulating the problem as a non-convex problem and finding the phase shifts of the WIT phase in a closed form. An alternating optimization algorithm was also introduced to find a solution to the sum-throughput maximization problem.

#### 6.1.2. Backscatter Communication

Backscattering is a promising technology for sensors that have low power consumption, such as IoT devices, as it consumes extremely low power. In this technique, a portion of the received signal is transmitted back to the source, which is primarily used for RF identification [43]. In [44], the authors investigated backscatter communication randomness using collision–resolution techniques. The authors concluded that combining several collision–detection techniques is promising for achieving significant gains.

#### 6.1.3. Optimal Power Control

In a WPSN, controlling the uplink transmit power is a crucial issue because a minimum amount of transmit power is required to maximize revenue. It can be easily comprehended that if the distance between the energy nodes and the sensor nodes is too large, it is very difficult to transmit the exact amount of energy according to sensor demands. In [69], the authors proposed a game-theory-based model in which the uplink transmit power was measured by each sensor in the game. Thus, the trajectory at which the optimal power can be achieved was reached upon reaching the Nash equilibrium, and the goal of maximizing the revenue was optimized.

#### 6.1.4. Energy Scheduling

As it is often difficult to predict the source of the energy supply, it is essential to manage the energy accordingly so that the efficient transfer of energy is ensured and the quality-of-service requirements are fulfilled. As resources such as radio sources and energy are scarce, it is more efficient to consider a distributed approach for transferring wireless energy, as it requires a very small amount of information exchange, which reduces the overhead at the network end. To mitigate this problem, [70] proposed a distributed algorithm that can adapt itself while selecting nodes according to their energy conditions. Subsequently, the nodes were scheduled to minimize the latency. The authors demonstrated that, compared to other state-of-the-art algorithms, the difference in latency was 2%.

Reinforcement learning-based approaches have also gained considerable attention in solving the energy scheduling problem in WPSNs. Addressing the issue of fast battery drainage remains a significant research challenge for WPSNs. In [70], the authors considered a Markov decision process, in which they considered energy consumption as well as the data queue to formulate WPT and data transmission problems. As it is a very common scenario, the sensors may not have sufficient information on the battery level and data queue length. This study emphasized that the field focuses on minimizing packet loss. The authors further extended their study when all the information was known by the base station.

#### 6.1.5. Energy Harvesting in Cooperative Networks

**Figure 8:**
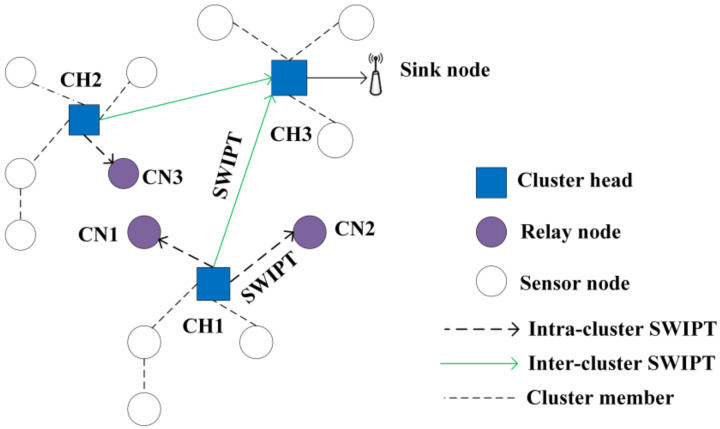
SWIPT-enabled clustered WSN.

In a wireless cooperative network, harvesting energy is crucial, as information relaying is possible in such networks. In a clustered WSN, the intermediate nodes make information relay possible by acting as relay nodes for transmitting information between multiple clusters. In such a scenario, allocating an optimal amount of power and determining the power-splitting ratio can significantly enhance the overall energy efficiency of the system. To address this issue, the authors in [71] considered SWIPT for charging relay nodes with limited energy in a clustered WSN, as shown in Figure 8. In the proposed scheme, the system can cooperatively transmit information between clusters. Moreover, because the relay nodes consume extra energy for forwarding data, these nodes can harvest energy as energy compensation. The authors formulated a cooperative transmission by considering the constraints of the minimum data rate, minimum energy, and maximum transmission power. The authors concluded that the power-splitting ratio was a determining factor for relay selection.

#### 6.1.6. Path Planning

Planning the path through which charging will be performed is another crucial area with a potential for improving the performance of the WPSN. The authors in [72] proposed such a system, in which the mobile-based energy station can select one potential path at a time section, and it is unable to change the path until it returns to the charging station. Accordingly, the authors demonstrated that the mobile energy station could traverse a planned path to charge the sensor in each area. The authors utilized a Markov decision policy to establish their model and used it to jointly optimize the planning of the path. They demonstrated that a significant performance improvement was obtained using their proposed methodology.

#### 6.1.7. Node Selection for Avoiding Eavesdropping

Avoiding potential eavesdropping is one of the most crucial design issues that still dominates the field of WPSNs. There have been several studies that deal with selecting dedicated nodes so that eavesdropping can be avoided in a network without consuming more energy. A joint selection policy for transmitting nodes in an eavesdropping environment was investigated in [73]. In this design, the nodes attempted to harvest energy from a power source using a passive eavesdropper. When the nodes were successfully charged, they were opportunistically scheduled to transmit data to the base station. The authors assumed that the energy-harvesting (EH) model follows a nonlinear model. The authors also formulated a power allocation problem concerning the transmission and jamming power. This study primarily focused on addressing the secrecy performance and node selection problem for IoT networks, particularly emphasizing energy harvesting. The node that achieved the best channel status aimed to transmit data, whereas the worst channel status was used for jamming the signal transmitted from the eavesdropper. This resulted in a minimization of the effect on the base station.

#### 6.1.8. Resource Allocation

Optimal resource allocation in WPSNs is another great technique to enhance the energy efficiency of WPSNs. The work in [74] emphasized energy efficiency using an optimal resource allocation policy. The authors considered a WPSN comprising a single antenna and several single-antenna sensors, where the sensors harvested energy from a hybrid access point. Upon receiving energy, the sensor could transmit information by utilizing nonorthogonal multiple access. The authors formulated an energy-efficiency maximization problem by considering the harvesting time and transmit power. A particle-swarm-optimization-based algorithm was proposed, owing to its stability and fast convergence.

#### 6.1.9. Computation Offloading

With advancements in technology, surveillance-related applications are growing significantly. Energy efficiency and minimal delay are the two crucial design metrics in such applications [75]. WSNs are widely used in fields such as environmental surveillance, healthcare, and security services [76]. In a WSN, performing a computation-intensive task is one of the most significant challenges, primarily because the sensors have energy constraints. Furthermore, most of the sensors being developed are delay-critical applications and have a limited battery life, which means that the sensors must complete their duty in the shortest possible time. In the case where any delay is introduced by a task, the overall task is delayed and the throughput is reduced, which is not expected at all. This is where computational offloading has an upper hand. The integration of mobile edge computing with a wireless sensor network allows the task to be offloaded to the nearest edge server. In [76], the authors presented a mobile edge computing-enabled wireless sensor network, where they attempted to solve the limited energy problem by introducing WPT. Accordingly, the authors proposed an algorithm to maximize the total number of processed bits. They divided the problem into several subproblems. Subsequently, they solved these subproblems by maximizing the ability of the unit cycle system.

### 6.2. Enabling Frameworks for Wireless Power Transfer

In the previous section, the key performance-enhancing techniques for WPSNs were outlined. In this subsection, the enabling frameworks that are needed to design and analyze WPT systems for sensor networks are emphasized. Since this field is still growing, there have been numerous techniques proposed from several disciplines, such as optimization concept, design of network, machine learning, system-level analysis, and signal processing techniques.

#### 6.2.1. Optimization Techniques

Optimization techniques have been used extensively for WPT applications. More specifically, a model of the WPT was first developed to develop an existing system, and optimization theories were utilized to solve the problem and verify its correctness. The advantages of such techniques are that optimal solutions can be found with reliable performance guarantees, and the results can be interpreted to obtain an understanding of the system design. In [77], the authors derived a successive convex approximation method to optimize the waveforms for WPT. The design operated adaptively for the frequency-selective channel and was a critical technique for obtaining the tradeoff between allocating the power in the carrier, which is the strongest, and allocating the power between several carriers. This resulted in the non-uniform allocation of power across *n* number of carriers.

Optimization techniques have also been proven to provide an optimal scheme in which the joint consideration of performance metrics is required. In a cognitive radio network, the authors in [78] aimed to maximize the rate of secondary users who aim to harvest energy utilizing downlink WPT by optimizing the transmit power and time allocation using the theory of convex optimization. However, most of these problems still require further investigation using advanced optimization techniques. Most of the existing problems fall into the criteria of a mixed-integer programming problem, which becomes very difficult to solve using traditional optimization techniques and requires a significant number of iterations to reach convergence. In such a use case, optimal transport theory [79] can significantly improve the modeling of energy-efficient systems.

#### 6.2.2. Machine Learning (ML) in WPT

Despite the significant advances in the field of communication in terms of several techniques, there are still several unknown systems in terms of behavior, such as the WPT system, in which the problem cannot be defined in a mathematical model form. Examples include the rectenna model, as well as the WPT architecture, while enhancing from either the system or the signal optimization perspective. Based on the above circumstances, ML techniques can play a crucial role, since this approach tends to come up with a target solution based on the underlying pattern of the training data instead of striving to model the problem mathematically. For example, in WPT, the data collected from circuit simulations can be fed into a deep neural network, and the optimal waveform can be found. More specifically, with supervised learning and training data consisting of input power, the load can be fed, and the best waveform can be classified as the output of the network [80,81,82]. The most important aspect of machine learning is that it generalizes well on unseen data, which is often not possible with traditional optimization techniques, which is why it has been used extensively in the literature [83] to solve communication-related problems, owing to its superiority. Recently, ML techniques have received considerable interest owing to the rich deep-learning libraries and the availability of intensive computation-friendly hardware. To detect the freshness of beverages by relying on WPT and near-field communication, a near-perfect accuracy of 96% was achieved using supervised machine learning for classifying milk freshness [84]. Moreover, in [85], the authors investigated a method for controlling tunable matching circuits in a WPT system, including transmitter coils, using pre-trained neural networks. The neural network produced a set of capacitance values, as well as the selection of a single transmitter among the others. ML techniques have also proven to be effective in partial discharge (PD)-based localization schemes, where WPT-enabled PD features are utilized to train supervised ML models that predict the location of PD afterward. This technique is crucial to more effectively maintain power systems [86]. However, a certain degree of performance uncertainty exists when using these techniques, as the accuracy of the learning models does not always provide a real picture [87]. Reinforcement learning (RL), another subsection of ML, is another crucial technique utilized in the literature [88]. For example, in the scenario of an unmanned aerial vehicle acting in a time-varying dynamic environment, the collection of data from such a complex environment is often impossible. In such a scenario, an agent interacts with the environment and decides to provide the maximum reward. In addition, RL can also deal with schemes that require adaptive behavior. Such a system was proposed in [89], where the authors sought to minimize the outage probability of information transfer by dynamically assigning channel resources in a wireless power communication system.

#### 6.2.3. Game-Theoretic Techniques

Game theory is another widely used technique in wireless communication that enables the modeling of interactions between several decision makers. Each decision maker comes up with an action that maximizes utility. These decision makers are known as players, and the moves they make are considered actions. In WPT, these three terms have been modeled differently in different studies. In [90], the authors considered a scenario in which a pair of sources and destinations competed to receive assistance from an energy-harvesting source. The authors proposed a strategy for allocating power by formulating a Nash equilibrium game. Accordingly, the authors in [91] proposed wireless information and power transfer, in which they considered the competition among relay nodes, which was formulated through a Nash game. The actions of the players were defined as the power-splitting ratio. To balance the transmission efficiency of information and the harvested energy of the relay, the authors in [92] studied a relay node with its energy-harvesting duty. The authors in [93] investigated energy transmission in a WPSN in a multi-antenna power beacon system where multiple users can harvest energy. In [94], the authors proposed an extension of the Stackelberg game, where all network information was available. However, the channel state and energy cost were unknown to the access point. Moreover, the authors in [95] proposed a robust Stackelberg game in which the channel state information was not perfect among the users and the power beacon.

It is evident that game-theoretic techniques can model the interactions between users and the energy-harvesting nodes, as well as address the model of cooperation between the source, relay nodes, and users. These characteristics motivated the present study to further investigate the achievement of enhanced system utility and better energy efficiency.

#### 6.2.4. Stochastic Geometry Methods

Stochastic geometry refers to the mathematical analysis of special random patterns. In wireless networks, this technique can model randomness in network topology [96]. In an ad hoc network, this problem becomes severe when the transmitter and receiver positions are randomly located at different locations. In such cases, maintaining the SNR of users becomes very challenging, owing to the presence of coupling interference [97]. In the WPSN scenario, owing to the random location of sensor nodes, it is very challenging to design the power transfer technique owing to many factors, such as determining the sensor node to serve, the number of beams to be generated, and the width of the beams. In [98], the authors addressed these challenges by focusing on the efficiency of energy transfer in large-scale sensor networks. The authors used stochastic geometry to derive metrics related to the distribution of received power at the sensor node. They concluded that the average received power increases with an increase in the density of the sensor nodes. The authors of [99] emphasized the tradeoff between information transfer and energy transmission in a WPCN using stochastic geometry. To achieve this tradeoff, the authors formulated a throughput maximization problem under successful information transfer constraints by jointly considering the portioning timeframe between the uplink and downlink phases and the transmit power of the sensor nodes. Furthermore, the authors considered two different types of wireless nodes: battery-free and battery-enabled wireless nodes. The authors of [100] considered a large-scale network consisting of transmitters and receivers, where a cluster of transmitting nodes jointly served a receiver. The locations of the transmitter and receiver were modeled using a Poisson point process. The authors analyzed the performance of the proposed system in terms of several metrics, such as the size of the cluster, rate of energy harvesting, and energy buffer size. The study concluded that an increase in the ratio of transmitter-to-receiver density is proportional to the size of the cluster.

## 7. New Challenges for Future Extension

Although extensive research has been conducted in the field of WPSNs, several challenges remain to be explored. Below, some new challenges are presented for future extensions that will carry out further research.

### 7.1. Multiple Sensor Nodes

It is observed that most existing works focus only on powering a single node. The implementation of charging large-scale sensor nodes is very complicated. In the presence of multiple sensor nodes, the power beacon needs to be able to equally distribute the amount of wireless power to each sensor node. As such, it is crucial to investigate how the power beacon can allocate equal power to multiple sensor nodes for enhanced network performance [101]. Several aspects are yet to be explored, such as time allocation, distribution of energy, and energy scheduling in terms of multiple sensor nodes [38].

### 7.2. Energy-Efficient WPSNs

Energy efficiency is considered as a crucial performance indicator for WPSNs. Taking the initial energy of sensors and the quality-of-service requirement into account, how to maximize the network’s energy efficiency is a challenging issue [102,103,104]. Although several studies have focused solely on energy efficiency, determining the optimal parameters for an energy-efficient WPSN in terms of the energy consumption of the power beacon remains a challenging task.

### 7.3. Duplex Mode

Typically, in WPSNs, the energy is transmitted from a dedicated energy source to the wireless sensor nodes via radiofrequency radiation. If the source is a dedicated energy transmitter, then it can transmit energy only to the sensor node. However, using a hybrid access point (HAP) that can be either half-duplex or full-duplex, both wireless information and energy can be transmitted. Thus, a full-duplex hybrid access point can outperform a half-duplex access point in association with the proper interference cancellation technique [105]. Thus, deciding the mode to operate is a challenging issue in WPSNs.

### 7.4. Multi-Hop WPSNs

Maximizing energy efficiency is a crucial issue for WPSNs. In a multi-hop communication-based sensor network, when one hop fails to maintain communication with the next hop, the entire communication is affected. In a multi-hop WPSN, powering the sensor nodes is very critical because of the extremely low efficiency of the traditional WPT technique [106]. In addition, the existing architecture of WPSNs only focuses on single-hop WPSNs, whereas multi-hop sensor nodes poses a risk of power loss since the number of hops may increase during transmission of the data [107]. The generalization of the energy-efficiency problem by jointly optimizing the transmission power and energy-harvesting time with the use of multi-hop remains a challenge.

### 7.5. Exchange of Information and Power

In the SWIPT technique, ensuring information and power transfer efficiency is of crucial importance due to exchanging both information and energy at the same time. However, because of the different characteristics of various devices in the network, the security issue and possible solution differs [12]. Therefore, much attention should be given to provide better service over the network.

### 7.6. IRS-Enabled WPSNs

Intelligent reflecting surfaces (IRSs) are deployed to enhance the overall performance of wireless energy transfer, as well as wireless information transfer. In the IRS-enabled WPSN, the IRS improves the overall performance by smartly adjusting the phase shift of each of the reflecting elements [42]. IRSs gather the energy signal, which is then reflected through the IRS planar array. Meanwhile, IoT devices utilize the harvested energy to transmit their information to the AP with the help of the IRS and time-division multiple access. It will be challenging to design an IRS-assisted WPSN when the cascaded channel state information (CSI) of the IRS-enabled IoT devices and the IoT device–IRS–AP channels are unknown [42].

### 7.7. UAV-Enabled Wireless Power Transfer

Recently, UAVs have emerged as a promising solution for serving a large number of ground users. Owing to their flexibility and high dynamicity, UAVs can flexibly fly in a three-dimensional (3D) area to provide energy to nearby ground devices [108]. However, in such a scenario, designing the trajectory of the UAV to ensure energy maximization is a crucial task, mainly because UAVs must deal with a dynamic environment. Techniques such as radio maps [109,110,111] and reinforcement learning [112] have been employed to update the UAV trajectory online. The availability of channel state information is another crucial aspect of this scenario. Designing a backscatter antenna for UAV networks is a potential solution [113] to ensure a longer network lifetime. In a multi-antenna multi-UAV scenario, the UAV must adopt a collaborative energy transmission strategy to maximize energy efficiency and cover the desired number of ground devices. However, owing to the limited battery lifetime, minimizing the communication delay is a crucial challenge in existing UAV communication, which can be solved by powering UAVs wirelessly [114]. To obtain channel estimation, additional time and energy are consumed to implement channel estimation/feedback. Hence, a tradeoff can be achieved between obtaining precise channel state information and minimizing time or energy consumption [115]. Millimeter-wave (mm-Wave)-enabled UAVs are another promising paradigm for which significant progress has been made in the existing literature. The mm-Wave signal being blocked by terrestrial infrastructure can be avoided by deploying UAVs because UAVs offer high mobility, which enables them to avoid obstacles in obtaining good channel conditions [116]. The authors in [117] considered an mm-Wave UAV communication system, where they emphasized improving the secrecy performance of UAV networks. The authors formulated the problem as a secrecy-rate maximization problem by considering the transmit power, power-splitting ratio, and UAV trajectory. They showed that the proposed approach can obtain an increased secrecy rate compared with other benchmark algorithms.

### 7.8. Millimeter-Wave Communication

Millimeter-wave communication is regarded as an emerging solution because it meets the prerequisites of future 5G networks [118]. To cope with the increased bandwidth-intensive applications, mm-Wave enables transferring the transmission of data to an unused spectrum with sufficient bandwidth, such as mm-Wave. To achieve significant network performance, wireless power transfer should be considered jointly with mm-Waves. In [119], the authors considered mm-Wave-enabled WPS, where energy was transferred from an access point to selected sensors utilizing the beamforming technique. The sensors used the harvested energy to power the uplink transmission. The authors considered a large-scale approach, in which they used the Poisson point process to characterize the randomness of the sensors. The authors considered an application based on event monitoring and distributed information to prove the performance of the proposed location selection scheme. From the considered application, the authors concluded that the event estimation was more accurate when the cell radius was small.

Furthermore, mm-Wave-enabled SWIPT systems have gained significant attention as another promising approach, owing to the extended energy coverage provided by the mm-Wave power transfer. Research in this field is still insufficient because of the shortage of practical hardware. To design such systems, several metrics, such as power allocation, beamforming, and power splitting, should be jointly considered to ensure the performance of mm-Wave SWIPT systems [120].

## 8. Conclusions

In this study, a survey of wireless power transfer in WPSNs has been presented. A novel taxonomy of various WPT techniques reported for WPSNs so far has been provided. The WPT techniques have been extensively compared with each other in terms of their advantages, limitations, innovative features, and performance metrics. In addition, the important techniques for enhancing WPT efficiency and the enabling frameworks for WPT in WPSNs have also been discussed because they need to be considered to enhance the performance of WPT in WPSNs. Lastly, the crucial future research directions have been discussed, which will motivate further research efforts in WPSNs.

By utilizing wirelessly harvested energy, WPSNs can significantly improve overall network performance, reliability, and system throughput. Although existing techniques provide significant outcomes for extending the system lifetime, it is essential to design WPSNs by considering system scalability and energy-transfer efficiency. To achieve the next step in future wireless communications, WPSNs will become an inevitable building block for obtaining prolonged system operation in the future.

## Figures and Tables

**Table 2:** Summary of WPT techniques in terms of advantages and limitations.

WPT Technique	Advantages	Limitations
Capacitive coupling [58]	Power transfer range up to kilowatts Power can pass through metal objects (however, in some cases it is not possible) The use of metal plates reduces costs Suitable for small applications	Limited efficiency Power transmission distance is insufficient
Inductive coupling [58]	Power transfer range is up to kilowatts Efficiency can be achieved up to 90% (but, in exceptional cases, even higher than 90%) Applications requiring power from low power to high are viable candidates	Power transmission distance is insufficient The nearby metals generate eddy current loss, which affects the nearby area
Microwave power transfer [58]	Transmission distance is very large (up to several km) Appropriate for applications running on mobile devices Can transfer power up to several kilowatts; however, this poses a biological threat for humans and animals	Inefficient for high-power-intensive application Implementation is complicated
Laser power transfer [58]	Transmission distance is very large Flexible and appropriate for mobile devices Up to several kilowatts of power can be transmitted over a considerable distance	Efficiency is low Line of sight to the receiver is necessary
Ultrasonic power transfer [59]	Higher penetration depth and shorter wavelength Can travel through electrically conductive materials	Generates heat Efficiency near 45% in implantable devices

**Table 3:** Comparison of WPT techniques in terms of operational characteristics.

WPT Technique	Operational Range	Frequency	Strength	Mobility	Multicast	Safety
Capacitive coupling [19]	Low	Up to MHz	High	Absent	Absent	High
Inductive coupling [19]	Low	Up to MHz	High	Absent	Absent	High
Microwave power transfer [19]	High	Up to GHz	Low	Present	Present	Medium
Laser power transfer [19]	High	Greater than THz	High	Absent	Absent	Medium
Ultrasonic power transfer [59]	Low	Greater than 20 KHz	Low	-	-	Medium (Due to the directed sound beam in long-term exposure)

**Table 4:** Summary of SWIPT Techniques.

SWIPT Technique	Main Ideas
Separate receiver	Individual receiver for information decoding and energy decoding Perform both information decoding and energy decoding independently Served by a common antenna transmitter
Power-splitting	Perform energy harvesting and information decoding simultaneously A tradeoff can be achieved between energy harvesting and information decoding compared to other methods The power-splitting ratio can be optimized for each receiver
Time-switching	The receiver periodically switches between energy harvesting and information decoding Both energy harvesting and information decoding cannot be performed simultaneously It is possible to optimize the waveform for energy harvesting and information decoding
Antenna-switching	Antenna-switching complexity is low Performance may not be satisfactory due to hardware limitations Easy to implement Can be utilized to enhance the performance of separate receiver architecture

**Table 5:** Comparison of existing WPT Technologies in terms of the main idea, advantages, and limitations.

WPT Technique	Main Idea	Advantages	Limitations
Ref. [45]	Demonstrated a method for wirelessly transferring power through a metal barrier by incorporating capacitive coupling and inductive coupling.	The magnetic flux density near the edge is higher.	Output power is affected by the variation of distance.
Ref. [46]	Proposed the design of a lightweight and energy-efficient multi-MHz IPT system.	The proposed system can wirelessly power a battery-less drone, and magnetic field exposure to human tissue is much lower.	The coupling range is less.
Ref. [47]	Demonstrated an electromagnetic rectifying surface for overcoming the deterioration of an antenna.	Using the proposed design, the radiated power can be received without any reflection.	Transmission efficiency declines as the angle deviation increases.
Ref. [48]	Presented a distributed laser charging approach to solve transferring power over meter-level distance for IoT devices.	Provides both theoretical and practical insight into designing a distributed laser charging technique.	Photovoltaic panel efficiency is inefficient, providing only 50% efficiency.

**Table 6:** Comparison of existing WPT techniques in terms of the evaluated performance metrics, objective, and outstanding features.

WPT Technique	Evaluated Performance Metrics	Goal of the Study	Innovative Features	Evaluation Tool
Ref. [45]	Output power, magnetic flux density.	Maximize output power and magnetic flux density.	Incorporates a metal barrier.	-
Ref. [46]	End-to-end power transfer efficiency, coupling factor.	Achieve power transfer efficiency at the optimal load.	Allows operating at a maximum coupling and minimum power.	SPICE
Ref. [47]	Transmission efficiency, incident angle, input power.	Maximize transmission efficiency.	Resolves the directivity deterioration problem by using an electromagnetic rectifying surface.	CST
Ref. [48]	Wavelength of the laser, distance of transmission, photovoltaic cell temperature.	Maximize the power transmission efficiency.	Proposed design is verified using practical performance metrics.	MATLAB

**Table 7:** Comparison of existing SWIPT techniques in terms of the main idea, advantages, and limitations.

SWIPT Technique	Main Idea	Advantages	Limitations
Ref. [36]	Demonstrated two receiver designs for SWIPT systems to investigate the performance of wireless power transfer (WPT).	Proposed a strategy to achieve an optimal transmission scheme for obtaining different tradeoffs.	Did not consider fading channel with partial channel information.
Ref. [49]	Proposed a conflict-solving transmission schedule initialization algorithm.	Proposed an approach to maximize the energy efficiency of WPSN.	The energy transfer ratio decreases when the network radius is large.
Ref. [50]	Designed power-splitting ratio for obtaining optimal SWIPT performance.	Designed a power-splitting receiver by considering the error in the channel estimation.	Did not consider multi-antenna transmission scenario.
Ref. [51]	Proposed a dynamic antenna-switching method for reducing the complexity of SWIPT systems.	The proposed method can be utilized in multi-user interference scenarios.	Considered that the antennas are randomly ordered (ignoring the antenna selection policy).

**Table 8:** Comparison of existing SWIPT techniques in terms of the evaluated performance metrics, objective, and outstanding features.

SWIPT Technique	Evaluated Performance Metrics	Optimization Objective	Innovative Features	Evaluation Tool
Ref. [36]	Information rate–energy tradeoff.	Maximize transported energy efficiency and information rate to the energy harvesting and information decoding.	Can be extended to investigate the influence of background noise and co-channel interference.	-
Ref. [49]	Network radius, average cooperative energy transfer ratio, sink broadcasting power, number of nodes.	Maximize energy efficiency.	Jointly considered optimal resource allocation and energy efficiency.	-
Ref. [50]	Optimal power splitting, normalized energy-harvesting constraint.	Maximize the ergodic capacity by meeting the energy-harvesting constraints.	Proposed work can be directly utilized in time-division multiple-access schemes.	MATLAB
Ref. [51]	Outage probability.	Minimize the outage probability.	Proved the impact of channel condition and network geometry on channel allocation.	MATLAB

**Table 9:** Comparison of existing WPCN techniques in terms of the main idea, advantages, and limitations.

WPCN Technique	Main Idea	Advantages	Limitations
Ref. [52]	Investigated a channel-learning strategy for multi-user MIMO systems.	Proposed a method that can handle a large number of energy receivers.	Further improvements can be made using more than one feedback bit.
Ref. [53]	Studied a WPCN where energy and information transfer are coordinated by one multi-access point.	Considered a multi-antenna access point and single-antenna user.	Considered a single access point with multiple users. Not suitable for large-scale users.
Ref. [54]	Demonstrated cooperative behavior of a user for optimizing throughput of WPCN.	Proposed solution can effectively enhance both user fairness and network throughput.	Did not consider more than two users.
Ref. [55]	Studied the placement optimization problem of energy and information access points.	Proposed method can minimize the network deployment cost, ensuring guaranteed performance.	Co-channel interference was ignored.

**Table 10:** Comparison of existing WPCN techniques in terms of design approach and main idea.

WPCN Technique	Evaluated Performance Metrics	Optimization Objectives	Innovative Features	Evaluation Tool
Ref. [52]	Normalized error of harvested power and estimated matrix norm, average harvested power.	Maximize the weighted sum energy.	Proposed a single feedback bit approach to determine the increase or decrease in the harvested energy.	MATLAB
Ref. [53]	Number of iterations, number of active transmit antennas.	Maximize the minimum throughput among all users.	Jointly considered downlink and uplink time allocation.	MATLAB
Ref. [54]	Throughput, transmit power, distance ratio.	Maximize the weighted sum rate of two users.	Jointly optimized time and power allocation in the network for both uplink and downlink.	-
Ref. [55]	Net energy-harvesting rate, CPU time, deployment cost.	Minimize the network deployment cost.	Jointly considered placement problem of energy node and access point.	MATLAB

Note: “-” means that the information is not specified in the corresponding literature.

## Data Availability

Not applicable.
